# Lei’s formula attenuates osteoarthritis mediated by suppression of chondrocyte senescence via the mTOR axis: *in vitro* and *in vivo* experiments

**DOI:** 10.18632/aging.205582

**Published:** 2024-02-23

**Authors:** Xing Zhou, Wen-Kai Li, Chen Zhuang, Xing-Chen Zhou, Xue-Fei Zhao, Yu Pan, Wen-Xuan Guo, Yi-Wen Yang, Cen-Zhuo Sheng, Zhe-Fei Xie, Jin-Sheng Yu, Yi-Xuan Chen, Li-Kang Wang, Tian-You Ma, Kang-Xiang Zhu, Ke-Meng Xiang, Ru-Jie Zhuang

**Affiliations:** 1 The First School of Clinical Medicine, Zhejiang Chinese Medical University, Hangzhou, Zhejiang, China; 2 Alberta Institute, Wenzhou Medical University, Wenzhou, Zhejiang, China; 3 The Third School of Clinical Medicine (School of Rehabilitation Medicine), Zhejiang Chinese Medical University, Hangzhou, Zhejiang, China; 4 Department of Orthopaedics, The First Affiliated Hospital of Zhejiang Chinese Medical University (Zhejiang Provincial Hospital of Traditional Chinese Medicine), Hangzhou, Zhejiang, China; 5 Taizhou Traditional Chinese Medicine Hospital, Taizhou, Zhejiang, China; 6 Quzhou Hospital of Traditional Chinese Medicine, Quzhou, Zhejiang, China; 7 Quzhou TCM Hospital at the Junction of Four Provinces Affiliated to Zhejiang Chinese Medical University, Quzhou, Zhejiang, China

**Keywords:** Lei’s formula, osteoarthritis, chondrocyte, senescence, mTOR axis

## Abstract

Lei’s formula (LSF), a traditional Chinese herbal remedy, is recognized for its remarkable clinical effectiveness in treating osteoarthritis (OA). Despite its therapeutic potential, the exact molecular mechanisms underlying LSF’s action in OA have remained enigmatic. Existing research has shed light on the role of the mTOR signaling pathway in promoting chondrocyte senescence, a central factor in OA-related cartilage degeneration. Consequently, targeting mTOR to mitigate chondrocyte senescence presents a promising avenue for OA treatment. The primary objective of this study is to establish LSF’s chondroprotective potential and confirm its anti-osteoarthritic efficacy through mTOR inhibition. *In vivo* assessments using an OA mouse model reveal substantial articular cartilage degeneration. However, LSF serves as an effective guardian of articular cartilage, evidenced by reduced subchondral osteosclerosis, increased cartilage thickness, improved surface smoothness, decreased OARSI scores, elevated expression of cartilage anabolic markers (Col2 and Aggrecan), reduced expression of catabolic markers (Adamts5 and MMP13), increased expression of the chondrocyte hypertrophy marker (Col10), and decreased expression of chondrocyte senescence markers (P16 and P21). *In vitro* findings demonstrate that LSF shields chondrocytes from H_2_O_2_-induced apoptosis, inhibits senescence, enhances chondrocyte differentiation, promotes the synthesis of type II collagen and proteoglycans, and reduces cartilage degradation. Mechanistically, LSF suppresses chondrocyte senescence through the mTOR axis, orchestrating the equilibrium between chondrocyte anabolism and catabolism, ultimately leading to reduced apoptosis and decelerated OA cartilage degradation. LSF holds significant promise as a therapeutic approach for OA treatment, offering new insights into potential treatments for this prevalent age-related condition.

## INTRODUCTION

Osteoarthritis (OA), a complex condition impacting skeletal cartilage, involves the breakdown and degeneration of cartilage tissue [1, 2]. This chronic and painful disease lacks a specific treatment plan, often resulting in disability and financial strain for patients. Predictions indicate that OA will become the 4th most disabling disease by 2050 [3]. The absence of specialized treatment places a significant psychological and physical burden on patients, coupled with substantial healthcare costs [4]. Chondrocytes, the primary cell type in articular cartilage, are pivotal in maintaining cartilage homeostasis by secreting and synthesizing the extracellular matrix (ECM) [5]. Senescence of chondrocytes, induced by factors like aging, stress, abnormal stimulation, telomere shortening, oxidative stress, and chromatin abnormalities, triggers the activation of the DNA damage response (DDR). This results in cell cycle block through pathways such as p53/p21/pRb/E2F and p16/pRb/E2F, leading to cellular senescence [6, 7]. Characteristic features of chondrocyte senescence encompass altered cellular morphology, telomere shortening, increased expression of p21, p16, and p53, elevated levels of reactive oxygen species (ROS), and enhanced senescence-associated β-galactosidase (SA-β-gal) activity [8]. Senescence-induced OA progression involves the release of a senescence-associated secretory phenotype (SASP) containing inflammatory factors, chemokines, growth factors, and proteases, contributing to inflammation, bone redundancy formation, and ECM degradation. The synovial fluid of OA patients shows elevated SASP factors, fostering a vicious cycle by driving further senescence of surrounding cells. Senescence also stimulates the expression of MMP families, including heightened activity of MMP13 and ADAMTs-5, responsible for degrading ECM proteins in cartilage [9–11]. Evidence indicates that senescent chondrocytes (SnC) aggregate with age and are significantly upregulated in human OA cartilage compared to healthy controls [8]. Excessive SnC exacerbates articular cartilage destruction, whereas SnC clearance attenuates subsequent senescence and tissue damage to surrounding cells, thereby slowing OA progression [12].

mTOR, a serine/threonine protein kinase, plays a critical role in cell proliferation, growth, and differentiation [13]. Positioned at the intersection of multiple intracellular signaling pathways, mTOR closely regulates apoptosis, autophagy, and senescence [14, 15]. Upstream signaling pathways converging at mTOR include the PI3K/mTOR signaling pathway, AMPK/mTOR signaling pathway, and mTOR activation through specific amino acids [16, 17]. Upon organismal stimulation, PI3K is activated, leading to the phosphorylation of Akt by activated PI3K. Akt transmits signals to downstream targets, ultimately activating mTOR, inhibiting cell autophagy, and regulating the expression of apoptosis-related genes [18]. Inhibitors of the PI3K/AKT/mTOR signaling axis exacerbate oxidative stress damage by increasing ROS accumulation and decreasing NAPDH levels [19]. Notably, anti-apoptosis-related protein genes are up-regulated in senescent cells, including members of the phosphatidylinositol-3-kinase/protein kinase B (PI3K/Akt) signaling pathway. This upregulation promotes autophagy of senescent cells, effectively controlling the degradation of ECM to slow down chondrocyte senescence, thereby protecting articular cartilage and inhibiting OA progression [20]. Studies have shown increased mTOR expression in articular chondrocytes of mice with KOA. Furthermore, mTOR-specific knockout in mice led to a significant upregulation of autophagy signaling in cartilage, resulting in the inhibition of articular cartilage degeneration and reduction of apoptosis [21–23].

Traditional Chinese medicine (TCM) holds a significant position in conservative treatment options for osteoarthritis (OA). Lei’s formula (LSF) is derived from Lei’s medical treatise “Treatise on Chronic Diseases” and has been widely utilized in OA treatment. LSF includes the Chinese medicinal pair Duhuo-Qianghuo, known for their anti-inflammatory and analgesic effects, as demonstrated in previous studies [24–28]. Table 1 summarizes the herbs present in LSF. Duhuo and Qianghuo, constituents of LSF, are known for dispelling wind, dampness, and relieving pain, while Fangfeng has properties that disperse wind, relieve epidemics and dampness, and ease pain. The anti-inflammatory and analgesic effects of Fangfeng can effectively alleviate osteoarthritis symptoms, as per traditional use [27, 29, 30]. Furthermore, osthol, an active chemical ingredient in LSF, has been identified to reduce oxidative stress damage to chondrocytes and delay cellular senescence. These findings align with our previous clinical observations demonstrating LSF’s ability to improve osteoarthritis symptoms [31, 32]. However, the specific pathological mechanism by which LSF acts on OA remains unclear.

**Table 1:** The list of herbal names in LSF.

**Chinese name**	**Botanical name (The Plant List)**	**Part used**	**Weight (g)**
Qianghuo	*Notopterygium incisum* Ting ex H.T.Chang	root	10
Fangfeng	*Saposhnikovia divaricate* (Trucz.) Schischk.	root	10
Duhuo	*Heracleum hemsleyanum* Diels	root	10
Xixin	*Asarum heterotropoides* F.Schmidt	root	3
Cangzhu	*Atractylodes lancea* DC.	rhizome	10
Gancao	*Glycyrrhiza uralensis* Fisch. ex DC.	root	3

To replicate cellular senescence and simulate the pathogenesis of OA, this study employed H_2_O_2_ to induce chondrocytes, creating a model of oxidative stress injury. This model triggered an imbalance in cellular catabolism and invoked a senescence response. The primary objective of this study was to investigate whether LSF exhibits anti-chondrocyte anabolic-catabolic imbalance, anti-apoptotic, and anti-senescence effects both *in vitro* and *in vivo*. Additionally, the study aimed to determine whether LSF achieves these effects through the mTOR signaling axis.

## RESULTS

### HPLC analysis of bioactive compounds in LSF

**Figure 1:**
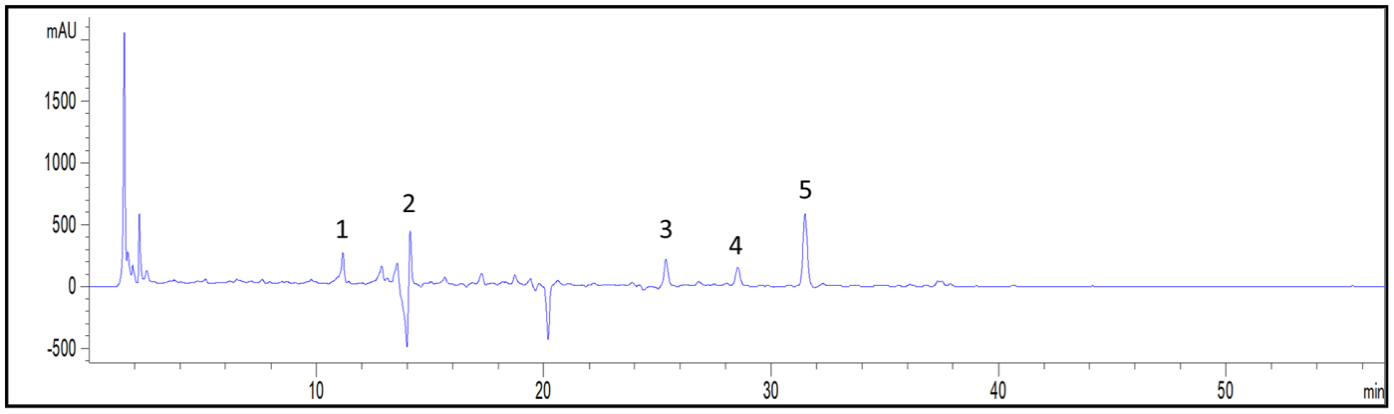
**Representative chromatogram of major compounds in LSF.** 1. Cimicifugoside; 2. Notopterol; 3. Compound Glycyrrhizin; 4. Atractylodin; 5. Osthole.

The herbal raw materials of LSF were sourced from the First Affiliated Hospital of Zhejiang Chinese Medicine University. LSF lyophilized powder was obtained following the processes of decoction, concentration, and freeze-drying. High-performance liquid chromatography (HPLC) was employed to determine the contents of representative chemical components in LSF, ensuring their stability and reproducibility. The HPLC analysis revealed the presence of five main chemical constituents in LSF: 1. Cimicifugoside; 2. Notopterol; 3. Compound Glycyrrhizin; 4. Atractylodin; 5. Osthole (Figure 1). Additionally, the contents of these major chemical components were quantified by comparing them with the corresponding standards, as presented in Table 2.

**Table 2:** Main compounds of LSF.

**No**	**Compounds**	**Chemical formula**	**Contents (mg/g)**
1	Cimicifugoside	C_22_H_28_O_11_	39.87
2	Notopterol	C_21_H_22_O_5_	31.55
3	Compound Glycyrrhizin	C_42_H_62_O_16_	16.68
4	Atractylodin	C_13_H_10_O	6.91
5	Osthole	C_16_H_16_O_3_	67.79

### Effect of LSF on the viability of chondrocytes cultured with or without H_2_O_2_

**Figure 2:**
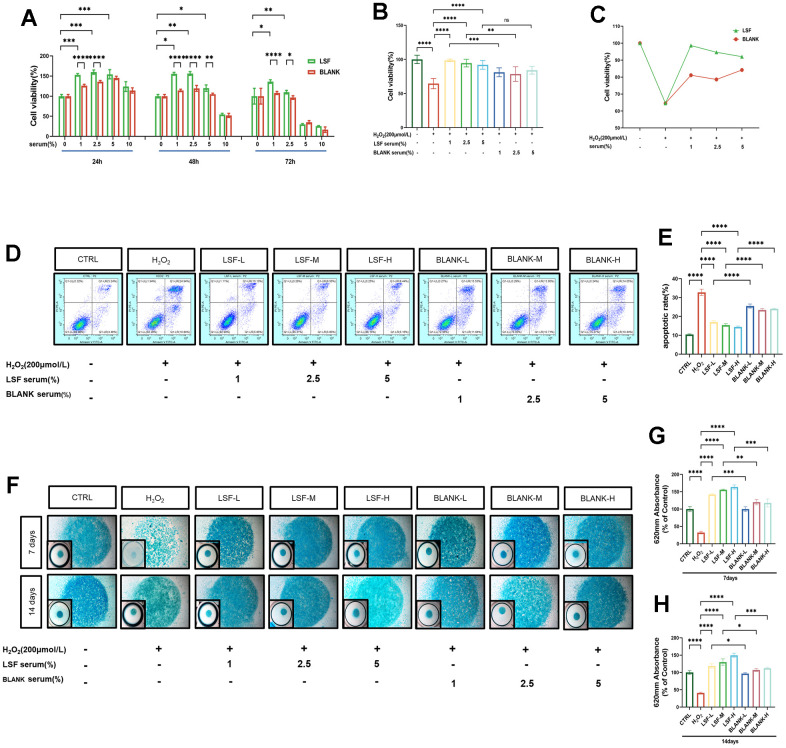
**Effect of LSF on chondrocyte viability in H_2_O_2_-stimulated chondrocytes.** (**A**) Effects of different concentrations of LSF- containing serum acting for different times on chondrocyte viability as determined by CCK8. (**B**, **C**) Chondrocytes were pre-treated with LSF-containing serum at screened gradient concentrations before being given the appropriate concentration of H_2_O_2_ for incubation, as assessed by CCK8. (**D**, **E**) The treated chondrocytes were double-stained with Annexin V-FITC/PI and then subjected to flow cytometry to assess the effect of gradient concentration of LSF-containing serum on the apoptotic rate of chondrocytes. (**F**–**H**) The treated chondrocytes were cultured in microspheres (7 and 14 days) and stained with Alcian blue to assess the differentiation ability of chondrocytes, and the intensity of the blue color represented the differentiation ability of chondrocytes, and the absorbance was detected by an enzyme marker set at a wavelength of 620 nm. All data represent mean ± SD. Compared to the control group, *P < 0.05, **P < 0.01, ***P < 0.001, ****P < 0.0001; compared to the H_2_O_2_-stimulated group, ****P < 0.0001; compared to the same concentration of blank serum group, *P < 0.05, **P < 0.01, ***P < 0.001, ****P < 0.0001.

To determine the effective and non-toxic concentration range of LSF on chondrocytes, varying concentrations of LSF-containing serum (0%, 1%, 2.5%, 5%, 10%) were co-incubated with primary mice chondrocytes for 24, 48, and 72 hours. Cell viability was assessed using the CCK-8 assay. As illustrated in Figure 2A, LSF demonstrated no cytotoxicity at concentrations below 5% for all time points (24, 48, and 72h). Moreover, cell viability was notably improved compared to the blank serum group at the same concentration, displaying a dose-dependent trend of LSF efficacy at 24 hours. Therefore, for subsequent experiments assessing the effect of LSF on H_2_O_2_-stimulated chondrocytes, gradient concentrations of 1%, 2.5%, and 5% LSF-containing serum were utilized (Figure 2A).

Mice primary chondrocytes were pre-treated with the aforementioned LSF-containing serum concentrations (0%, 1%, 2.5%, 5%) for 24 hours. Subsequently, they were stimulated with H_2_O_2_ at concentrations as previously determined for 24 hours (details shown in Supplementary Figure 1). Cell viability was assessed using the CCK-8 assay. As depicted in Figure 2B, 2C, the viability of H_2_O_2_-stimulated chondrocytes markedly decreased. LSF exhibited a dose-dependent reversal of this trend, displaying superior efficacy compared to the same concentration of the blank serum group.

### LSF limits apoptosis and promotes chondrogenic differentiation in H_2_O_2_-stimulated chondrocytes

To elucidate the mechanisms by which LSF enhances viability in H_2_O_2_-stimulated chondrocytes, we conducted apoptosis rate detection using flow cytometry with Annexin V-FITC/PI double-stained chondrocytes. Our results demonstrated a significant increase in the apoptotic rate of chondrocytes upon H_2_O_2_ stimulation. However, pretreatment with LSF significantly reduced the apoptotic rate, even surpassing the same concentration of the blank serum group (Figure 2D, 2E).

Furthermore, we investigated chondrocyte differentiation in mice primary chondrocytes after treatment with differentiation-inducing culture medium for 7 and 14 days. Alcian blue staining was employed to visualize the acidic mucopolysaccharides in chondrocytes. The staining showed a notable reduction in blue coloration, indicating a decline in chondrocyte acidic mucopolysaccharides in the H_2_O_2_-stimulated group, whether for 7 or 14 days. Importantly, LSF treatment mitigated this trend and significantly enhanced chondrogenic differentiation compared to the same concentration of the blank serum group (Figure 2F, 2G).

### LSF modulates H_2_O_2_-stimulated chondrocyte anabolism and inhibits cellular senescence

**Figure 3:**
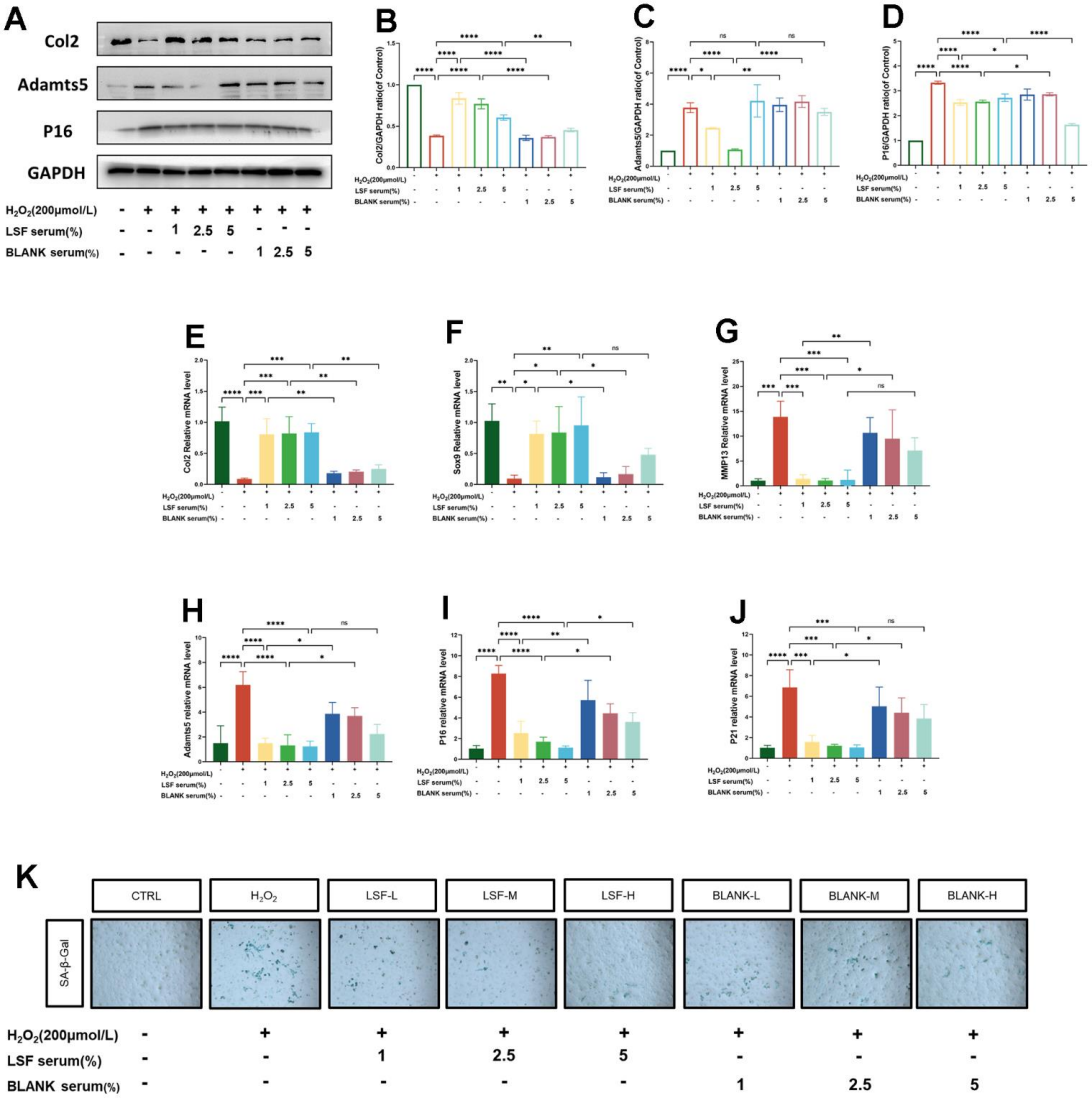
**LSF regulates chondrocyte anabolic-catabolic homeostasis and inhibits chondrocyte senescence.** (**A**–**D**) Western blotting assessed the expression of Col2, Adamts5 and P16 proteins in chondrocytes. (**E**–**J**) qRT-PCR was performed to detect the expression of Col2, Sox9, MMP13, Adamts5, P16 and P21 in chondrocytes. (**K**) Cellular senescence was assessed by SA-β-gal staining. All data represent mean ± SD. Compared to the control group, **P < 0.01, ***P < 0.001, ****P < 0.0001; compared to the H_2_O_2_-stimulated group, *P < 0.05, **P < 0.01, ***P < 0.001, ****P < 0.0001; compared to the same concentration of blank serum group, *P < 0.05, **P < 0.01, ****P < 0.0001.

Previous studies have identified Col2, Sox9, Adamts5, and MMP13 as critical markers in regulating the balance between anabolism and catabolism in chondrocytes. Conversely, elevated expressions of P16, P21, and increased β-galactosidase activity are characteristic features of cellular senescence. In our qPCR analysis (Figure 3E, 3F), we observed a significant reduction in mRNA expression levels of Col2 and Sox9 in the H_2_O_2_-stimulated group. Additionally, the results (Figure 3G–3J) revealed a noteworthy increase in mRNA expression levels of Adamts5, MMP13, P16, and P21, all of which were effectively reversed by LSF. To delve deeper into LSF’s role in regulating anabolic catabolism and inhibiting cellular senescence in H_2_O_2_-stimulated chondrocytes, we conducted Western blotting to measure the protein expression of Col2, Adamts5, and P16. As depicted in (Figure 3A–3D), the protein expression of Col2 was significantly reduced in the H_2_O_2_-stimulated group. Conversely, protein levels of Adamts5 and P16 were markedly increased. Notably, LSF treatment effectively counteracted these alterations, aligning with the qPCR findings. Additionally, SA-β-gal staining revealed a considerable increase in blue coloration in the H_2_O_2_-stimulated group, indicating elevated β-galactosidase activity. LSF treatment demonstrated a significant improvement in this aspect (Figure 3K).

### LSF modulates H_2_O_2_-stimulated chondrocyte synthetic catabolic phenotype associated with the mTOR signaling axis

**Figure 4:**
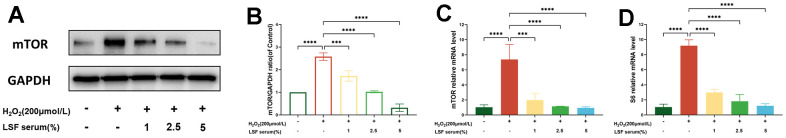
**LSF inhibits the H_2_O_2_-stimulated oxidative stress injury in chondrocytes and modulates the mTOR signaling pathway.** (**A**, **B**) Gradient concentrations of LSF-containing serum were pre-treated with chondrocytes before being given the appropriate concentration of H_2_O_2_ for incubation, and the expression of mTOR was detected by Western blotting experiments. (**C**, **D**) qRT-PCR was performed to detect the expression of mTOR and S6 in chondrocytes processed with LSF and H_2_O_2_. All data represent mean ± SD. Compared to the control group, ****P < 0.0001; compared to the H_2_O_2_-stimulated group, ***P < 0.001, ****P < 0.0001.

mTOR, a protein kinase intricately linked to cell proliferation, growth, and differentiation, is known to play a pivotal role in regulating apoptosis, autophagy, and cellular senescence. Thus, it becomes crucial to explore whether LSF’s regulation of chondrocyte anabolic-catabolic balance and inhibition of cellular senescence is linked to the mTOR signaling pathway. We initiated this exploration by assessing the expression of mTOR in H_2_O_2_-stimulated chondrocytes via Western blotting. Strikingly, mTOR proteins exhibited significantly heightened expression. However, in chondrocytes pre-treated with LSF, this trend was dose-dependently reversed by LSF (Figure 4A, 4B). To substantiate this finding, we conducted qPCR to measure the expression of mTOR and its downstream S6 gene. Encouragingly, the results mirrored the Western blotting findings (Figure 4C, 4D). Collectively, LSF holds promise in inhibiting cellular senescence and regulating chondrocyte anabolic-catabolic homeostasis through the mTOR signaling axis.

### Activation of the mTOR signaling axis disrupts chondrocyte anabolic balance and promotes cellular senescence

**Figure 5:**
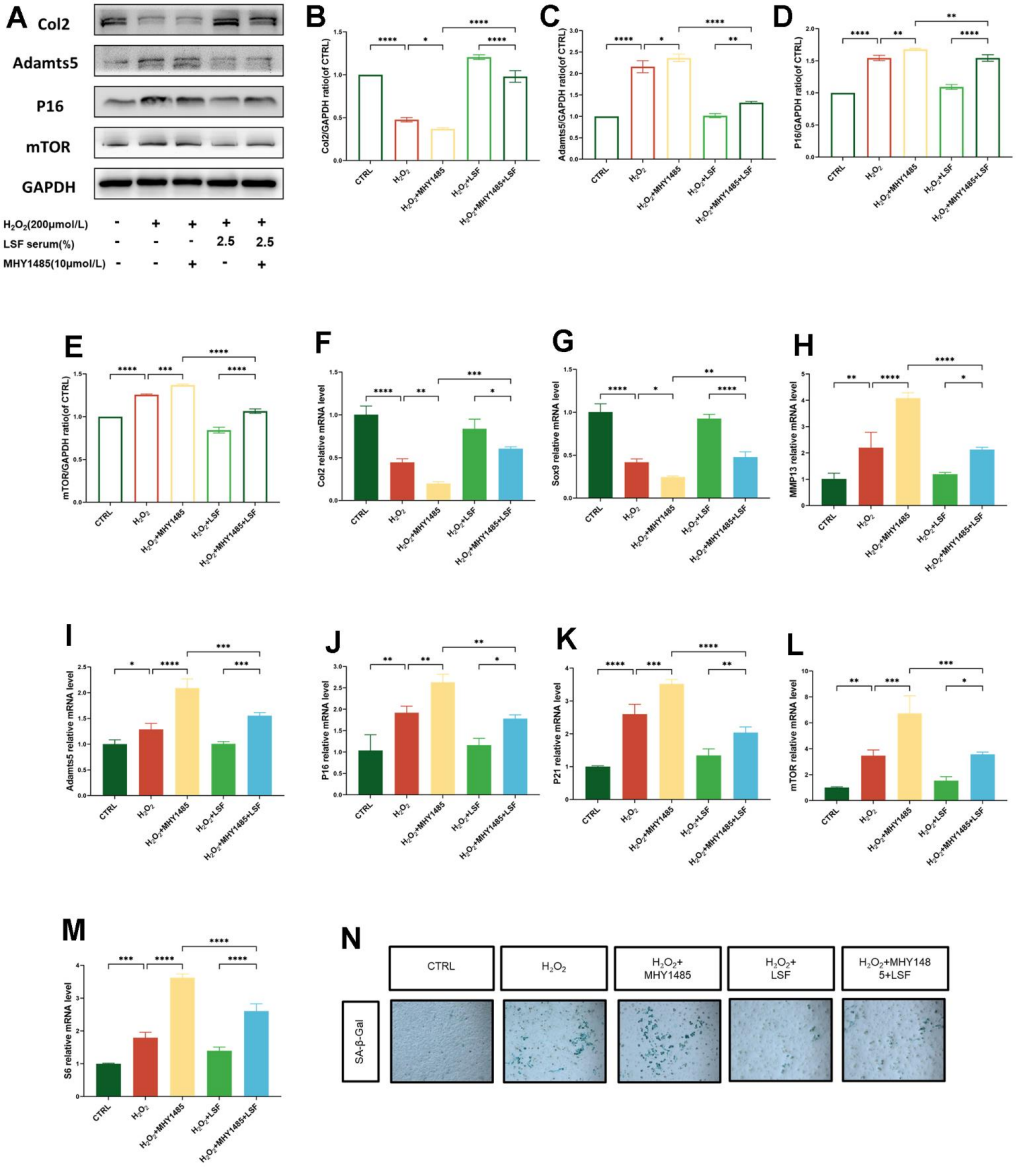
**Activation of mTOR exacerbates H_2_O_2_-stimulated chondrocyte senescence and anabolic catabolic disorders that can be reversed by LSF.** (**A**) Western blotting for protein expression of Col2, Adamts5, P16 and mTOR in chondrocytes processed with H_2_O_2_ (200 μmol/L) and MHY1485 (10 μmol/L). (**B**–**E**) Quantitative results of Western blotting of the 5 groups of proteins. (**F**–**M**) Expression of Col2, Sox9, MMP13, Adamts5, P16, P21, mTOR, and S6 in chondrocytes processed with H_2_O_2_ (200 μmol/L) and MHY1485 (10 μmol/L) was detected by qRT-PCR. (**N**) Cellular senescence was assessed by SA-β-gal staining. Compared to the control group, *P < 0.05, **P < 0.01, ***P < 0.001, ****P < 0.0001; compared to the H_2_O_2_-stimulated group, *P <0.05, *P < 0.01, ***P < 0.001, ****P<0.0001; compared to the H_2_O_2_-stimulated combined MHY1485 group, *P < 0.05, **P < 0.01, ***P <0.001, ****P < 0.0001.

To elucidate the impact of mTOR on H_2_O_2_-stimulated chondrocyte anabolic catabolism *in vitro*, we treated chondrocytes stimulated with H_2_O_2_ with the mTOR activator MHY1485 (10μM). Remarkably, Col2 expression was significantly suppressed in the H_2_O_2_+MHY1485 conditioned group, accompanied by increased expression of Adamts5, P16, and mTOR (Figure 5A–5E). Subsequently, we assessed the mRNA expression levels of genes associated with anabolic catabolic phenotype, senescence phenotype, and the mTOR signaling pathway in chondrocytes using qPCR. As depicted in Figure 5F–5M, the mRNA expression of Col2 and Sox9 was notably suppressed in cells subjected to H_2_O_2_+MHY1485 conditioning. Conversely, the mRNA expression of Adamts5, MMP13, P16, P21, mTOR, and S6 was heightened in the same group. Importantly, this trend was reversed in chondrocytes pretreated with LSF. Additionally, we performed SA-β-gal staining, which displayed a substantial increase in blue coloration and β-galactosidase activity in cells subjected to H_2_O_2_+MHY1485 conditioning. Strikingly, LSF ameliorated this effect (Figure 5N). Collectively, these findings suggest that suppressing the activation of the mTOR signaling axis could potentially reduce cellular senescence and mitigate osteoarthritis-associated chondrocyte catabolism, promoting an anabolic phenotype.

### LSF suppresses chondrocyte senescence via the mTOR pathway to mediate chondrocyte anabolic-catabolic homeostasis

**Figure 6:**
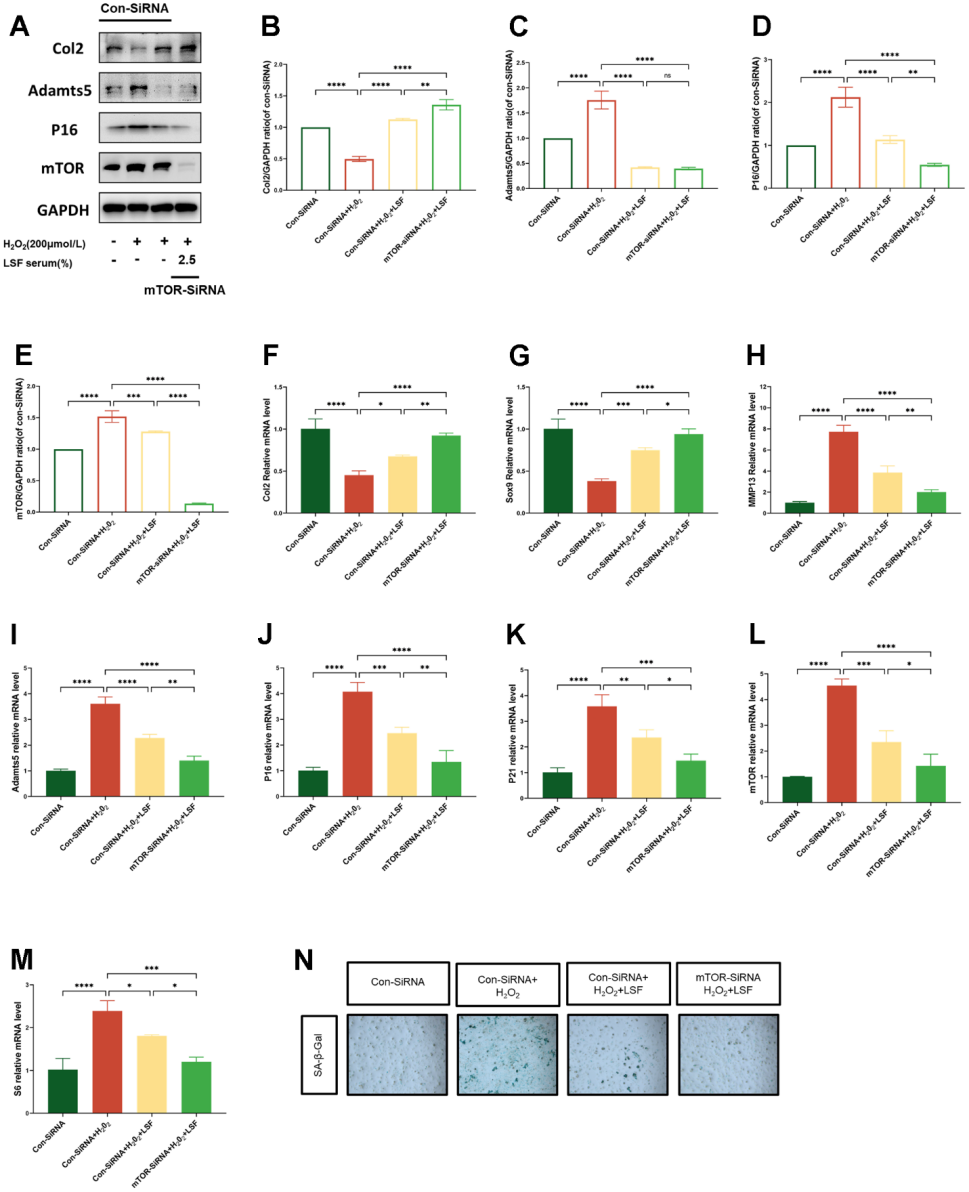
**mTOR-SiRNA contributes to LSF suppression of H_2_O_2_-stimulated chondrocyte senescence and promotion of anabolism.** (**A**) Westen blotting for protein expression of Col2, Adamts5, P16 and mTOR in lentivirus-transfected chondrocytes stimulated by H2O2 (200 μmol/L). (**B**–**E**) Quantitative results of Western blotting of the 4 groups of proteins. (**F**–**M**) Expression of Col2, Sox9, MMP13, Adamts5, P16, P21, mTOR, and S6 in lentivirus-transfected chondrocytes stimulated with H2O2 (200 μmol/L) was detected by qRT-PCR. (**N**) Cellular senescence was assessed by SA-β-gal staining. Compared to Con-SiRNA control group, ****P < 0.0001; compared to the H_2_O_2_-stimulated group, *P < 0.05, **P < 0.01, ***P < 0.001, ****P < 0.0001; compared to the mTOR-SiRNA group, *P < 0.05, **P < 0.01, ***P<0.001, ****P < 0.0001.

To validate the protective effect of LSF-triggered mTOR pathway on H_2_O_2_-stimulated chondrocytes, we transfected mTOR-siRNA into chondrocytes. Subsequently, Western blotting was employed to assess the expression of mTOR, Col2, Adamts5, and P16 in chondrocytes post transfection. As illustrated in Figure 6A–6E, mTOR protein expression in the mTOR-siRNA group was significantly reduced. Notably, Col2 expression was significantly up-regulated, and the expression levels of Adamts5 and P16 were diminished in the cells following LSF pre-treatment and H_2_O_2_ stimulation. Furthermore, qPCR analysis revealed elevated mRNA expression of Col2 and Sox9, whereas mRNA expression of Adamts5, MMP13, P16, P21, mTOR, and S6 was suppressed (Figure 6F–6M). Additionally, SA-β-gal staining was employed, and the results indicated a substantial reduction in blue coloration, signifying a decrease in β-galactosidase activity in the mTOR-siRNA group (Figure 6N). These comprehensive findings underscore that LSF exerts chondroprotective effects by mitigating cell senescence and resisting chondrocyte catabolism, ultimately promoting anabolism through the suppression of the chondrocyte mTOR pathway.

### LSF attenuates the progression of cartilage degeneration in a DMM-induced mice model

**Figure 7:**
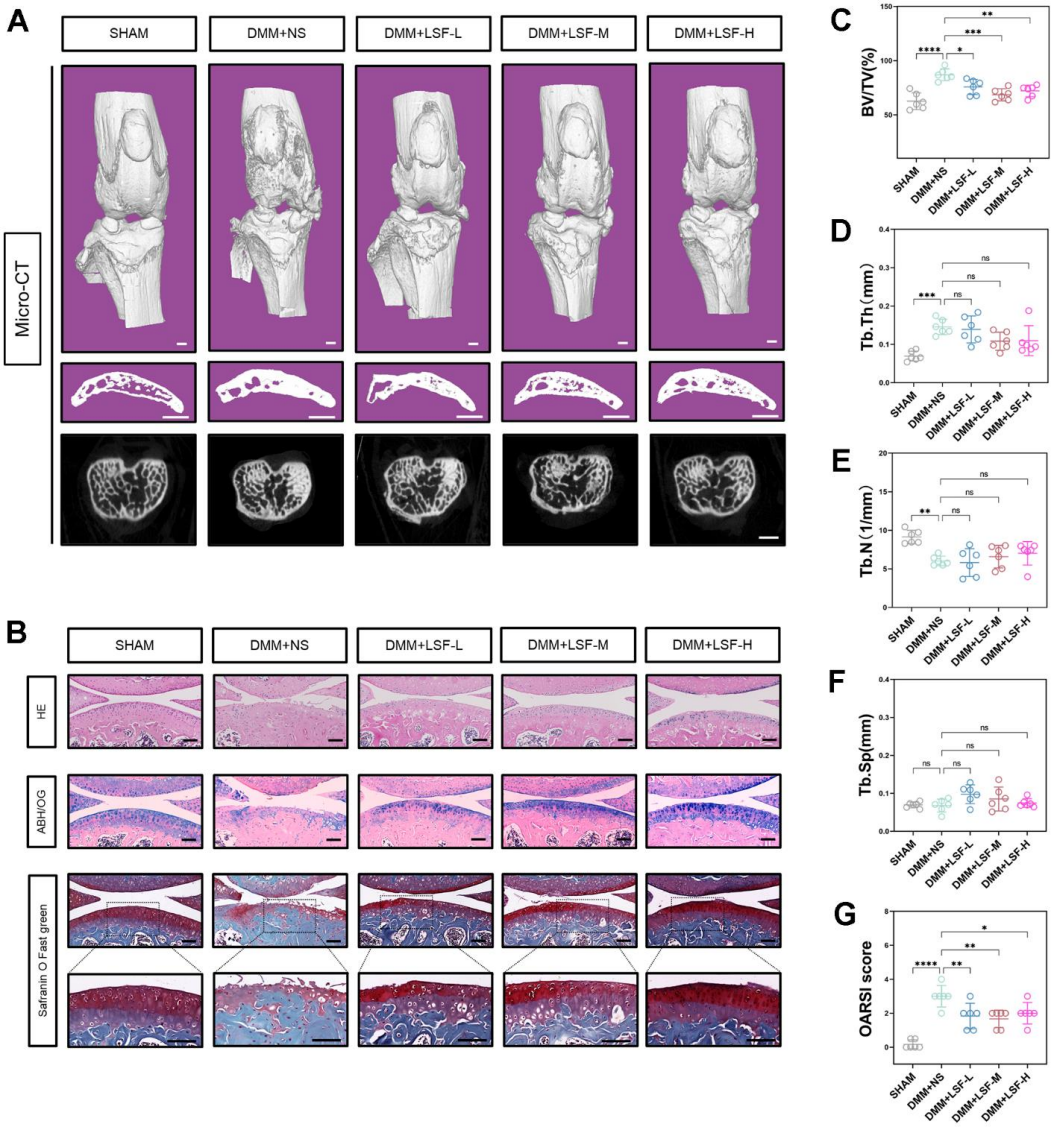
**LSF attenuates the deterioration of OA in DMM mice.** (**A**) Representative images of Micro-CT 3D reconstruction of mice knee joints. Mice were randomly assigned to treatment groups with different concentrations of LSF (0.08, 0.17, and 0.35 g/kg) after DMM surgery and received LSF via intragastric administration for 4 weeks. All mice were executed at 8 weeks postoperatively, Scale bar: 1 mm. (**B**) Typical images of mouse knee joints stained with HE, ABH/OG and Safranin O Fast green staining after pathologic sectioning. Scale bar: 50 μm. (**C**–**F**) Quantitative data of BV/TV, Tb.Th, Tb.N, and Tb.Sp of the subchondral bone in each group of mice at 8 weeks postoperatively. (**G**) OARSI score of the cartilage. All data represent mean ± SD. Compared to the SHAM group; **p ≤ 0.01, ***P < 0.001, ****P < 0.0001; compared to the DMM group, *P<0.05, **P < 0.01, ***P < 0.001.

To investigate the potential cartilage-protective effect of LSF *in vivo*, we established a destabilization of the medial meniscus (DMM)-induced mice model. Micro-CT scans and subsequent 3D reconstructions revealed a significant improvement by LSF in subchondral osteosclerosis and bone redundancy formation in the DMM-induced osteoarthritis mice. Quantitative results from the Micro-CT scans and the 3D reconstructions, including BV/TV, Tb.Th, Tb.N, and Tb.Sp, are presented in Figure 7A, 7C–7F. Histological examination of knee joints using HE, ABH/OG, and Safranin O-Fast Green staining depicted that the articular cartilage in the DMM-induced model mice exhibited structural disorganization, thinning, and defects. LSF intervention effectively rescued these histopathological changes, ameliorating cartilage degradation, increasing cartilage thickness, and promoting surface smoothness, as demonstrated in Figure 7B. The OARSI score was employed to quantify the staining results, illustrated in Figure 7G.

**Figure 8:**
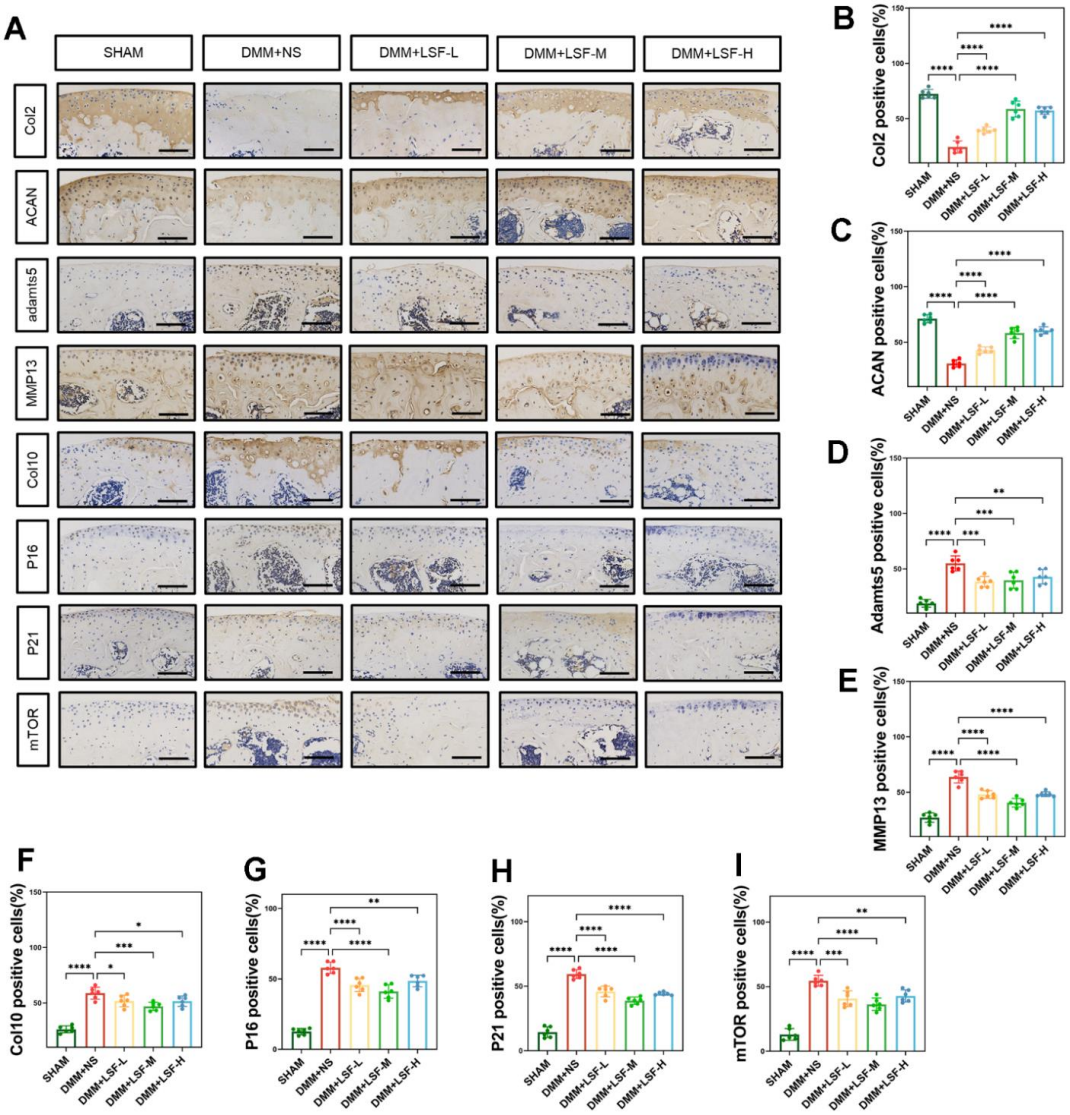
**LSF ameliorates the progression of OA and inhibits mTOR expression in the mice DMM model.** (**A**) Immunohisto- chemical typical images of pathological sections of mice knee joints from different experimental groups (SHAM, DMM, DMM+LSF-L (0.08 g/kg), DMM+LSF-M (0.17 g/kg), and DMM+LSF-H (0.35 g/kg)), which were examined for the detection of Col2, ACAN, Adamts5, MMP13, Col10, P16, P21 and mTOR expression in mice cartilage. (Scale bar. 50 μm). (**B**–**I**) Quantitative results of immunohistochemical staining in 5 groups of mice. All data represent mean ± SD. compared to the SHAM group; ****P < 0.0001; compared to the DMM group, *P < 0.05, **P < 0.01, ***P < 0.001, ****P < 0.0001.

Furthermore, immunohistochemistry revealed noteworthy findings. The expression levels of Adamts5, MMP13, Col10, P16, and P21 were significantly elevated, while the expression of Col2 and ACAN was decreased in the DMM group compared to the SHAM group. Interestingly, LSF administration reversed these trends, significantly enhancing cartilage sclerosis and mitigating the extent of cartilage degeneration in the DMM-induced mice, as displayed in Figure 8A–8I.

## DISCUSSION

OA is a prevalent and debilitating degenerative disease that primarily affects middle-aged and elderly individuals, carrying a high incidence rate [33, 34]. This disease imposes a substantial burden on individuals, families, and society due to its persistent and painful nature, as well as the lack of a definitive cure [3, 35, 36]. The current treatment landscape for OA is challenging, given the scarcity of highly effective drugs. Conservative treatments, including oral non-steroidal anti-inflammatory drugs, physical therapy, and functional exercises, often provide only partial relief to patients [37], For patients with moderate to advanced OA or those who do not respond to conservative treatments, surgical interventions, such as joint replacement surgery, are often recommended [4], However, these surgeries can be highly invasive and add to the physical, psychological, and economic burden on patients and their families [17, 38, 39]. Therefore, there is a pressing need to explore more potent drugs to effectively treat OA. Lei’s formula (LSF), an integral component of traditional Chinese medicine, incorporates Duhuo-Qianghuo pair and a variety of effective chemical components, including Osthole and Notopterol [24–26, 28, 32, 40]. Previous studies have confirmed its anti-inflammatory and antioxidative properties, making it a promising candidate for OA treatment. However, the specific pathogenic mechanism underlying the cartilage-protecting effect of LSF remains to be elucidated. The progression of OA primarily results from the disruption of chondrocyte and extracellular matrix homeostatic balance. Oxidative stress induced by H_2_O_2_ disrupts this balance by promoting chondrocyte senescence, accelerating the degeneration of cartilage and aggravating OA [41, 42]. This study was designed to investigate how LSF, in an *in vitro* setting, can inhibit the mTOR signaling pathway, thereby reducing H_2_O_2_-induced chondrocyte senescence and stabilizing the dynamic balance of extracellular matrix synthesis and catabolism. Additionally, in an *in vivo* setting, LSF was shown to mitigate cartilage degeneration in a DMM-induced OA mouse model.

The mTOR signaling pathway plays a pivotal role in mammalian senescence. Extensive research suggests that inhibiting mTOR enhances cellular autophagy, reduces the senescence-associated secretory phenotype (SASP), mitigates oxidative stress accumulation, and aids in removing damaged proteins and organelles, ultimately slowing down senescence [43–47]. Specifically, tuberous sclerosis complex-1 (TSC1) and TSC-2 typically form a dimeric complex that acts as an inhibitor of the small GTPase Rheb (Ras-homolog enriched in brain), a protein necessary for mTOR activation. This complex inhibits mTOR function under normal conditions [48–50]. However, when the organism is stimulated, PI3K is activated, leading to the phosphorylation of Akt. Activated Akt, in turn, phosphorylates Ser939 and Thr1462 of TSC-2, inhibiting the formation of the TSC-1/TSC-2 complex. Consequently, this removes the inhibitory effect on Rheb, resulting in mTOR activation. The activation of mTOR leads to decreased expression of autophagy-related proteins Beclin1 and LC3 and an increase in senescence-associated secretory phenotypes (SASP), such as inflammatory factors and proteases [18, 51–54]. This cascade of events can accelerate the transition to OA. The findings of this study demonstrate that LSF effectively reduces oxidative stress damage and inhibits cellular senescence in H_2_O_2_-stimulated chondrocytes, likely through the suppression of the mTOR signaling axis.

In the realm of *in vivo* investigations, the mouse model of DMM-induced OA is widely recognized for its ability to closely mimic the chronic OA process observed in humans. Given this, our study conducted *in vivo* experiments on mice induced with DMM, assessing them through Micro-CT and histopathologic staining. The results revealed that mice treated with LSF demonstrated significant reductions in subchondral osteosclerosis, decreased osteoid formation, and improved articular cartilage thickness and smoothness. This improvement was further reflected in the reduced OARSI score. Immunohistochemistry results affirmed that LSF had a positive impact, reducing chondrocyte hypertrophy, promoting anabolism, inhibiting catabolism, retarding chondrocyte senescence, and ultimately mitigating cartilage degradation. This holistic effect aided in delaying the progression of osteoarthritis. The outcomes of both *in vivo* and *in vitro* experiments were consistent and aligned, reinforcing the notion that LSF plays a crucial role in retarding the progression of OA. The study underscores the potential of LSF as a viable therapeutic option in managing and mitigating the effects of osteoarthritis.

We acknowledge that our current study has certain limitations. Firstly, our focus was primarily on investigating the effects of LSF on the mTOR signaling axis, without a detailed exploration of the responses of upstream and downstream proteins. This leaves the in-depth mechanism of LSF’s effects not fully elucidated. Future research should aim to delve deeper into the mTOR pathway by examining the responses of proteins both upstream and downstream, providing a comprehensive understanding of LSF’s mechanism of action. Secondly, our *in vivo* study was confined to a DMM-induced mice model. The absence of knockout mice limited our ability to thoroughly investigate the *in vivo* mechanism of LSF’s effects. Incorporating knockout mice in future studies could offer a more comprehensive understanding of LSF’s impact. Furthermore, we acknowledge that the specific active chemical components within the LSF compound remain unidentified. Future studies should prioritize isolating and identifying these key active chemical constituents, using advanced analytical techniques. This will enable a precise understanding of the components primarily responsible for the observed effects on osteoarthritis. Addressing these limitations in future research will enhance our comprehension of LSF’s potential in treating osteoarthritis and facilitate the development of targeted and effective therapeutic interventions.

## CONCLUSIONS

In summary, our research underscores the promising potential of LSF in mitigating the progression of osteoarthritis (OA). Through the inhibition of the mTOR signaling pathway, LSF effectively suppresses H_2_O_2_-induced chondrocyte senescence, resulting in a reduction of catabolism and promotion of anabolism in chondrocytes. Notably, our *in vivo* experiments validate these findings, demonstrating LSF’s capacity to mitigate cartilage degradation and decelerate OA progression in a mouse model induced by DMM. Looking forward, we anticipate further strides in this field. It is crucial to isolate and identify specific small molecule compounds from LSF, allowing for a more precise understanding of their mechanisms of action. These compounds have the potential to serve as targeted therapeutic agents, offering effective treatment for OA in the foreseeable future.

## MATERIALS AND METHODS

### Preparation of LSF and LSF-containing serum

LSF is a traditional Chinese medicine prescription comprising Qianghuo, Fangfeng, Duhong, Xinxin, Cangzhu, and Gancao. The botanical nomenclature was validated using http://www.worldfloraonline.org (Accessed on: 1 Oct 2023). Raw materials were sourced from the First Affiliated Hospital of Zhejiang Chinese Medicine University (Hangzhou, China), and the formula was prepared by mixing the aforementioned herbs and soaking them in four times their volume of water before boiling. The resulting herbal liquid was filtered and concentrated to 100mL using rotary evaporation at 55° C, followed by lyophilization in a freeze dryer to obtain the extract, which was stored at -40° C. For experimentation, approximately 20 Sprague-Dawley (SD) rats weighing approximately 250g were randomly divided into two groups: the LSF group and the blank group, each containing 10 rats. Rats in the LSF group were orally administered LSF, while those in the blank group received saline, twice daily for one week. Following euthanasia, blood samples were collected from the abdominal aorta, centrifuged to separate LSF-containing serum and non-containing serum, and stored at -40° C.

### High performance liquid chromatography (HPLC) analysis

LSF extract samples underwent separation using an Agilent 1260 system (Agilent, Waldbronn, Germany) employing a mobile phase composed of solvents A (pure water) and B (acetonitrile). The gradient elution program was structured as follows: 0-14 min with 78% A and 22% B, 14-15 min with a linear gradient elution of 22-30%B, followed by maintaining 30% B for 10 min from 15-25min. Subsequently, a linear gradient of 30-60% B was applied from 25-28 min, followed by maintaining 60% B for an additional 10 min. The flow rate was set at 1.0 mL/min, and a 20μL injection volume was used for sample analysis. The standards, including Notopterol, Isoimperatorin, Cimicifugoside, 4-O-beta-D-glucosyl-5-O-methylvisamminol, Asarinin, Columbianadin, Osthole, Atractylodin, Liquiritin, and Compound Glycyrrhizin, were procured from Chengdu Ruifenshi Biotechnology Co. (China).

### Extraction of mouse primary chondrocytes

Cartilage tissue from the knee joint of C57BL/6 mice was carefully dissected. The cartilage pieces were cut using ophthalmic scissors and underwent physical digestion. A 0.25% trypsin digestion was performed for 20 minutes, followed by PBS solution rinsing and incubation in DMEM medium containing 0.2% type II collagenase at 37° C in a water bath for 30-40 minutes with gentle agitation. Fetal bovine serum (FBS) was added to terminate the digestion. The mixture was then centrifuged at 1000 rpm for 5 minutes, and the supernatant was discarded. The remaining solution was centrifuged again at 1000 rpm for 5 minutes to obtain the digested chondrocytes. The obtained chondrocytes were cultured in DMEM/F12 containing 10% FBS and 1% antibiotics, and incubated in a humidified environment at 5% CO_2_ and 37° C. The culture medium was changed at regular intervals, and the chondrocytes were utilized for subsequent experiments.

### Reagents and antibodies

Fetal bovine serum (FBS) and DMEM/F12 medium were procured from CellMax (Beijing, China). The cytokine MHY1485 was sourced from MedChemExpress (USA). Primary antibodies targeting Col2 (1:200), Col10 (1:200), and MMP13 (1:200) were obtained from Abcam (Cambridge, UK), while the primary antibody against Aggrecan (1:200) was sourced from Proteintech (Wuhan, China). Primary antibodies against CDKN2A/p16INK4a (1:100) were purchased from Zenbio (Chengdu, China), and the primary antibody against Adamts5 (1:50) was acquired from HUABIO (Hangzhou, China). Primary antibodies against mTOR (1:200) were obtained from Cell Signaling Technology (USA).

### Cell culture and cell viability assay

Chondrocytes were cultured in DMEM/F12 supplemented with 10% FBS and 1% penicillin/streptomycin (CellMax, Beijing, China). Cell viability was assessed using the Cell Counting Kit-8 (CCK-8) (Bioss, Beijing, China). Chondrocytes were seeded in 96-well plates at a density of 2×10^4 cells per well following the instructions provided in the manual. After cell adhesion, varying concentrations of LSF-containing serum (1%, 2.5%, 5%, and 10%) and equivalent concentrations of blank serum were applied to the chondrocytes for 24, 48, and 72 hours. Another type of intervention involved using a gradient concentration of LSF-containing serum and the same concentration of blank serum (1%, 2.5%, and 5%) after pretreating chondrocytes for 24 hours, followed by replacement with 200 μM H_2_O_2_ and continuing the intervention for 24 hours. Subsequently, a 10% CCK-8 solution was prepared and added to the well plates, incubating for 2 hours in a 5% CO_2_ and 37° C incubator. Absorbance at 450 nm and 600 nm was measured using an enzyme marker (PerkinElmer, USA).

### Quantitative real-time PCR

Total RNA was isolated from the treated chondrocytes using TRIzol (Accurate Biology, Hunan, China). Subsequently, cDNA was synthesized using the Evo M-MLV Reverse Transcription Kit (Accurate Biology, Hunan, China). The cDNA was then amplified using a LightCycler 480II system (Roche, USA). Finally, the relative mRNA levels were determined using the 2^-ΔΔCT^ method [33]. Primer sequences utilized in this study can be found in Supplementary Table 1.

### Western blotting

Total protein was extracted from the treated chondrocytes using RIPA Lysis Buffer (CWBIO, Jiangsu, China) and Protease Inhibitor Cocktail (CWBIO, Jiangsu, China) as per the manufacturer’s instructions. Protein concentration was determined using the BCA Protein Assay Kit (Beyotime, Shanghai, China). The gel was prepared using the PAGE Gel Fast Preparation Kit (Epizyme, Shanghai, China), and proteins were separated by electrophoresis and subsequently transferred to a PVDF membrane (Millipore, USA). Incubation with 5% skimmed milk powder was carried out for 1 hour, and the gel was then incubated overnight at 4° C with primary antibodies such as GAPDH, Col2, Adamts5, p16, and mTOR. Subsequently, the membrane was incubated with the corresponding secondary antibodies for 1 hour at room temperature with agitation. Protein bands were finally detected using a protein imaging system (Proteinsimple, USA).

### Chondrocyte microsphere culture and Alcian Blue staining

Chondrocyte microsphere culture was employed to replicate the three-dimensional arrangement of chondrocyte morphology *in vitro*. A 5 μL chondrocyte suspension treated with the respective intervention was seeded into 48-well plates at a density of 1.5×10^7 cells per well. Following cell attachment, 500 μL of chondrogenic differentiation induction medium was added, consisting of insulin-transferrin-selenium (ITS) (Sigma, USA), 10ng/mL TGF-β3 (PeproTech, USA), 100 nM Dexamethasone (Sigma), 50 μg/mL VC (Sigma), 1 mM sodium pyruvate (Sigma), and 40 μg/mL proline (Sigma) in DMEM/F12. Half volume changes were performed daily. Incubation was continued for 7 and 14 days, respectively. Following incubation, the chondrocytes were stained using the Alcian Blue Staining Kit (Beyotime, Shanghai, China) to observe their differentiation ability.

### SA-β-gal staining

SA-β-gal staining, a hallmark of cellular senescence, was performed on treated cells to evaluate the degree of cellular senescence. The β-Galactosidase Staining Kit (Solarbio, Beijing, China) was employed for this purpose. Senescent chondrocytes were distinguished by a blue stain. Following the respective chondrocyte interventions in 12-well plates, 500 μL of β-galactosidase staining fixative was added to each well, and the cells were fixed at room temperature for 15 minutes. The staining working solution was prepared as per the supplier’s instructions. After fixation, the cells were rinsed three times with PBS, and 500 μL of staining solution was added to each well. Subsequently, the cells were incubated in a carbon dioxide-free incubator at 37° C overnight, and the wells were sealed with parafilm to prevent evaporation. Following incubation, observations and image documentation were carried out under an ordinary light microscope.

### Apoptosis detection

During chondrocyte digestion, cell cultures were collected, followed by centrifugation and cell counting. The cells were then resuspended using Annexin V-FITC conjugate. Specifically, 5μL of Annexin V-FITC (Solarbio, Beijing, China) and 10 μL of PI staining solution (Solarbio, Beijing, China) were added to the cell suspension. The cells were incubated for 15 minutes at room temperature. Subsequently, apoptosis in the cells was immediately detected using a flow cytometer (CytoFlexS, Beckman Coulter, USA).

### siRNA transfection

Chondrocytes were seeded in six-well plates at a density of 1×10^4 cells per well. After 24 hours, lentivirus containing the target siRNA sequence and an empty vector were introduced for transfection, which was halted after 16 hours. The culture medium was then replaced with complete medium and incubated for an additional 72 hours. Transfected cells were observed under a fluorescence microscope. Once the cell density exceeded 90%, puromycin at a concentration of 5.5μg/mL was employed to screen and eliminate untransfected cells. The remaining cells were passaged for expansion to be utilized in subsequent experiments.

### DMM-induced OA mice model

Eight-week-old C57BL/6 mice were randomly divided into five groups: SHAM, DMM, DMM+LSF-L (0.08g/kg), DMM+LSF-M (0.17g/kg), and DMM+LSF-H (0.35g/kg), each consisting of 6 mice. In the SHAM group, arthrotomy was performed only on the right knee joint. The DMM group involved the severance of the medial meniscus tibial ligament. Mice in the intervention groups were administered LSF intragastrically at graded concentrations (0.08, 0.17, and 0.35g/kg) twice daily for a duration of 4 weeks. After 8 weeks post-surgery, all mice were euthanized, and their knee joints were collected for subsequent histopathological examination.

### Micro-CT scanning and histological analysis

The right knee joints were obtained from euthanized mice and fixed by immersion in 4% paraformaldehyde. Following fixation, Micro-CT scanning was performed using a Skyscan1176 instrument (Bruker micro CT N.V, Kontich, Belgium). Subsequently, the joints were decalcified with EDTA for a period of 2 weeks. The decalcified samples were then paraffin-embedded, and 4-μm-thick pathology sections were prepared. Histological staining techniques including HE staining, ABH/OG staining, and Safranin O-Fast Green staining were employed. The degree of cartilage degeneration was evaluated using the Osteoarthritis Research Society International (OARSI) scoring system. For immunohistochemistry, the pathology sections were dried overnight, followed by dewaxing and rehydration. Antigen retrieval and blocking procedures were carried out, followed by the addition of primary antibodies and an overnight incubation. The subsequent day involved incubation with secondary antibody conjugate, followed by color development using DAB. The stained sections were observed under a microscope and photographed.

### Statistical analysis

All experimental data were analyzed using SPSS version 20.0. Measurements conforming to a normal distribution were presented as mean±standard deviation (mean±s), and intergroup comparisons were conducted using the t-test. For data not following a normal distribution, values were expressed as M (Q1-Q3), and the nonparametric rank sum test was employed for intergroup comparisons. The chi-square test was utilized for count data. A significance level of p < 0.05 was considered statistically significant. One-way ANOVA was applied for multi-group comparisons, and post hoc tests such as LSD or Dunnett’s T3 were used accordingly, with α=0.05 as the test level. A p-value less than 0.05 was considered statistically significant.

## Supplementary Material

Supplementary Figure 1

Supplementary Table 1
