# Supplementary material for: Lei’s formula attenuates osteoarthritis mediated by suppression of chondrocyte senescence via the mTOR axis: in vitro and in vivo experiments
Source: Aging (Albany NY). 2024 Feb 23;16(5):4250–69. doi: 10.18632/aging.205582 (PMC10968702; doi:10.18632/aging.205582)
Supplement: Supplementary Figure 1 [file aging-16-205582-s001.pdf]

SUPPLEMENTARY FIGURE

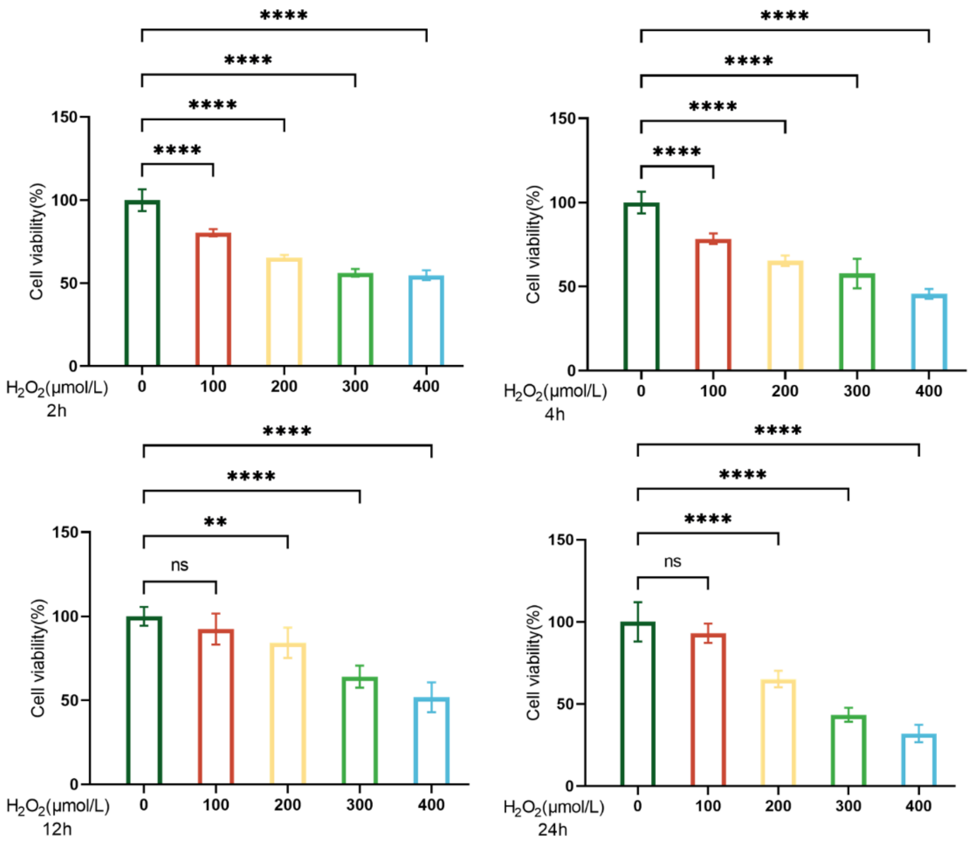

Supplementary Figure 1. Screening of H_2O-stimulated cellular oxidative stress model for optimal dose and duration of action. Different concentrations of H_2O_2 (0, 100, 200, 300, and 400 μM) stimulated the cells for 2h, 4h, 12h, and 24h, respectively, and it was observed that the cell viability was maintained at about 60%-70% at the concentration of 200 μM of H_2O_2, which could be used as the subsequent intervention concentration, as detected by CCK8.
