# Supplementary material for: Lei’s formula attenuates osteoarthritis mediated by suppression of chondrocyte senescence via the mTOR axis: in vitro and in vivo experiments
Source: Aging (Albany NY). 2024 Feb 23;16(5):4250–69. doi: 10.18632/aging.205582 (PMC10968702; doi:10.18632/aging.205582)
Supplement: Supplementary Table 1 [file aging-16-205582-s002.pdf]

SUPPLEMENTARY TABLE

Supplementary Table 1. Primer sequence.

Gene	Forward	Reverse
GAPDH	TGTGTCCGTCGTGGATCTGA	TTGCTGTTGAAGTCGCAGGAG
COL2A1	CCAGATTGAGAGCATCCGCA	ACTTTCATGGCGTCCAAGGT
SOX9	CCACCCCGATTACAAGTACCAG	CAGCGCCTTGAAGATAGCAT
MMP13	TTCTGGTCTTCTGGCACACG	TTGTAGCCTTTGGAACTGCTTG
ADAMTS5	GCTAAGGGCACAGGCTACTATG	CCGTCACATCCAGTTCTCACA
cdknla	TTGTCGCTGTCTTGCACT	AGAAGACCAATCTGCGCTTG
cdkn2a	GCTCTTCTGCTCAACTACGGT	CGATGTCTTGATGTCCCCGC
mTOR	GATCCATCTCGGCAACTTGACC	GCGTCAATCTTCCGAGCACT
RPS6	AACAGATTGCCAAGAGACGTA	AAGCCAATGAAAGATTCAAGGTC
